# A study of the incidence of milk fever in Jersey and Holstein cows at a dairy farm in Beatrice, Zimbabwe

**DOI:** 10.4102/jsava.v88i0.1457

**Published:** 2017-04-11

**Authors:** Bernard Chiwome, Erick Kandiwa, Borden Mushonga, Shepherd Sajeni, Gervais Habarugira

**Affiliations:** 1 School of Veterinary Medicine, University of Namibia, Namibia; 2 School of Animal Sciences and Veterinary Medicine, University of Rwanda, Rwanda

## Abstract

A 3-year prospective study investigated the incidence of milk fever in Jersey and Holstein cows at a commercial dairy farm in Beatrice, Zimbabwe. The overall incidence of milk fever at the farm was 7.98%. Incidence of milk fever in Jerseys (14.78%) was significantly higher (*p* < 0.05) than that in Holsteins (4.82%). Incidence of milk fever in Jerseys beyond their fourth parity (24.85%) was significantly higher (*p* < 0.05) than that in Jerseys in their second (5.90%), third (6.49%) and fourth (8.73%) parities. Incidence of milk fever in Holsteins beyond their fourth parity (8.29%) was significantly higher (*p* < 0.05) than that in Holsteins in their second (1.43%), third (1.82%) and fourth (2.91%) parities. No significant difference existed in milk fever incidences between the second, third and fourth parities in either Jersey or Holstein cows. Incidence of milk fever in Jersey cows producing over 6114 litres per 305-day lactation (27.07%) was significantly higher than that in Jerseys producing less than 6114 litres of milk per 305-day lactation (*p* < 0.05). Incidence of milk fever in Holsteins producing more than 9149 litres per 305-day lactation (10.49%) was significantly higher than that in Holsteins producing less than 9149 litres of milk per 305-day lactation (*p* < 0.05). No significant difference existed between incidences of milk fever between the first, second and third quartile producers (*p* > 0.05) in either Jersey or Holstein cows. This study confirms that the risk of developing milk fever is higher in Jerseys and also increases with increasing parity and higher levels of milk production in both breeds, thus advocating for special considerations when dairy cows fit these criteria.

## Introduction

Jersey and Holstein cattle make up the greater proportion (80%) of all the commercial dairy herds in the world. The Holstein-Friesland breed is by far the most popular breed, followed by the Jersey breed (Heins et al. 2008; Porter & Tebbit 2007; Weigel & Barlass 2003). Jersey cows have been widely selected for their high butterfat and Holstein-Friesland cows for large volumes of milk (Anderson et al. 2007; Burditt, Buchanan & Fitch 2000; Capper & Cady 2012). Extensive genetic manipulation of dairy cattle breeds over the past several decades for higher milk yields and butterfat has unfortunately been pursued at the expense of animal health and fertility (Hernandez 2013; Mulligan & Doherty 2008). According to Oltenacu and Broom (2010), breeding for productivity has also seriously overlooked welfare considerations for dairy animals. This blinkered focus on production has resulted in the increase of the incidence of production diseases or metabolic disorders such as milk fever, ketosis, hypomagnesaemia (Mulligan & Doherty 2008), fatty liver, lameness (Amory et al. 2008) and hypophosphataemia (Radostits et al. 2007). The transition period between late pregnancy and early lactation has been found to be the most significant risk period for the development of metabolic disorders in the dairy cow (Roche et al. 2013; Roche & Berry 2006), and this lasts for about 3 weeks post-partum (Mulligan & Doherty 2008). DeGaris and Lean (2008) suggested a longer period of 4 weeks post-partum as the period during which cows are most at risk for the development of metabolic disorders.

Milk fever (parturient paresis) is the most important and the most common metabolic disease of the transition cow (Mulligan et al. 2006b; Radostits et al. 2007). The disease has been the subject of over 50 years of extensive research in the dairy industry, and focus has been on the epidemiology, pathogenesis and risk factors (DeGaris & Lean 2008). Studies have shown periparturient health disorders not to be totally independent events, but rather a complex of interrelated disorders (DeGaris & Lean 2008; Drackley et al. 2005; Mulligan & Doherty 2008). Breed, nutrition, parity, milk production levels and genetic predisposition have been suggested as determining factors in the pathogenesis of milk fever (Mulligan et al. 2006b).

Milk fever is an acute to peracute, afebrile, flaccid paralysis of mature dairy cows that usually occurs within 48–72 hours of calving, although sometimes it may occur in late lactation (Radostits et al. 2007; Roche & Berry 2006). Researchers reported that most cows developed a subclinical hypocalcaemia in the periparturient period (LeBlanc 2010; Roche et al. 2013). However, it was only when blood calcium concentrations became insufficient to support neuromuscular function that it resulted in clinical signs (Roche & Berry 2006). Hypocalcaemia develops when concentrations of calcium in blood fall below 1.87 mmol/L. The normal serum concentration of calcium is 2.0 mmol/L – 2.5 mmol/L. Milk fever (clinical hypocalcaemia) is precipitated if the serum concentration drops further to below 1.625 mmol/L (Thirunavukkarasu et al. 2010). Hypocalcaemia at parturition results from the sudden increase in calcium requirements for colostrum and milk production (Mulligan et al. 2006b). Colostrum contains approximately four times more calcium than normal milk (Anderson et al. 2007; Tsioulpas, Grandison & Lewis 2007). Such sudden drainage of large amounts of calcium into milk can overwhelm the cow’s homeostatic mechanisms (Rizzo et al. 2008). Ideally, the cow adapts to this increased demand by increasing calcium absorption from the gastrointestinal tract and the mobilisation of calcium reserves from bone (Radostits et al. 2007). These processes are under the influence of parathyroid hormone, which catalyses the production of calcitriol from vitamin D_2_. The calcitriol stimulates increased gastrointestinal absorption of calcium and the mobilisation of calcium from bone (Goff 2008).

Despite a low mortality rate of 5%, it has been observed that the productive life of affected cows may be reduced (Jawor et al. 2012; Krehbiel 2014; Oltenacu & Broom 2010; Thirunavukkarasu et al. 2010). Affected cows have been found to be subsequently more susceptible to mastitis (Mulligan et al. 2006a), endometritis (Kimura, Reinhardt & Goff 2006), retained placenta and displaced abomasum (DeGaris & Lean 2008; Reinhardt et al. 2011). It has also been reported that between 4% and 28% of cows with milk fever relapse and eventually become downer cows (Goff 2008; Roche & Berry 2006; Stull et al. 2007). Economic losses result from mortalities, loss of production, increased culling rates and veterinary costs (Galvão 2012; Thirunavukkarasu et al. 2010).

On average, 5% – 10% of dairy cows succumb to clinical milk fever, with the literature suggesting that the incidence rate in individual herds can reach up to 80% of calving cows (DeGaris & Lean 2008). In New Zealand, clinical milk fever has been recorded to be as low as 3%, whereas subclinical milk fever is as high as 33% (Roche & Berry 2006). In some years, the incidence of milk fever in the United Kingdom has been recorded to be as high as 60% (Esslemont & Kossaibati 1996). Epidemiological studies show that the Jersey breed has the highest incidence of milk fever of all the dairy breeds followed closely by the Guernsey (Mulligan et al. 2006a). This occurrence is thought to be because of the high milk yield for the relatively small breeds. Jersey cows were reportedly at 2.25 times greater risk of milk fever than Holstein-Friesian cows (Lean et al. 2006). In another study, Goff (2008) suggested that Jersey cows were more predisposed to milk fever as they had less vitamin D receptors. Prapong et al. (2005) also showed that concentrations of the secretory enzyme Ca^2+^-ATPase in mammary tissue and milk fat globule membranes are directly proportional to the occurrence of milk fever. These breed differences in Ca^2+^-ATPase concentrations may also possibly contribute to breed differences in the incidence of milk fever.

There is clear evidence of the relationship between milk fever, lactation number and milk yield (DeGaris & Lean 2008; Jawor et al. 2012; Reinhardt et al. 2011). In fact, it has been reported that the risk of milk fever increases by 9% per lactation and that over-conditioned and high-producing cows were at a higher risk of developing milk fever (DeGaris & Lean 2008). In addition, it has been reported that there is a positive correlation between mastitis risk and high parity (Moosavi et al. 2014). It was likely because of the fact that despite the increasing requirement for calcium at parturition and the increased milk yield with each lactation, the ability to mobilise calcium from bone decreased with age (Horst 1986; Krehbiel 2014). High-yielding cows also had a higher energy demand to support milk production and were more likely to acquire a severe energy deficiency after calving than cows with lower yields. Therefore, higher milk yield has been associated with a greater risk for the development of metabolic diseases (Mulligan et al. 2006b).

Diagnosis of milk fever typically includes a history of recent calving, clinical signs of progressive ataxia, hypersensitivity and excitability to sternal recumbency, depression, dehydration and anorexia culminating in lateral recumbency, loss of consciousness, coma and even death if untreated (Radostits et al. 2007). The diagnosis of milk fever is usually confirmed by a positive response to treatment with calcium borogluconate (Radostits et al. 2007). The disease is confirmed by serum calcium assay (Reinhardt et al. 2011). Lean et al. (2006) used the dietary cation difference equation method in meta-analytical studies to predict the occurrence of milk fever.

Early treatment of the recumbent cow suffering from milk fever is essential to prevent necrosis of the tissues of the side on which the animal is lying, which is known to commence within 4 hours of recumbency (Goff 2008). The goal of treatment in milk fever is to restore the serum concentration of calcium sufficiently to support cellular function. It is achieved by intravenous administration of calcium salts such as borogluconate at a rate of 2 g/100 kg body weight (Goff 2008).

Prevention of milk fever involves several strategies including feeding of calcium-deficient diets in the late dry period, feeding of calcium-rich rations 3–4 days before parturition, vitamin D supplementation, reducing the dietary cation–anion difference and magnesium supplementation in the late gestation period (Goff 2008; Mulligan et al. 2006b).

The majority of epidemiologic studies on metabolic diseases of dairy cows have been carried out in the leading milk producing countries of the world with very little information on the comparative performance of Holstein and Jersey cows under similar feeding conditions in southern Africa (Muller & Botha 1998). Because there is a lack of data on the incidence of milk fever in Zimbabwean dairy herds, it was the aim of this study to investigate the effect of breed, parity and level of milk production on the incidence of milk fever on a mixed Holstein and Jersey commercial dairy herd over a period of 3 years.

## Methodology

A prospective evaluation of milk production and health records of a commercial dairy herd on a farm located in Beatrice, 50 km south of Harare composed of 471 Holstein-Friesian cows and 181 Jersey cows, was carried out. This particular farm (supplying about 50 000 L of milk weekly to Dairy Board Limited, Harare, Zimbabwe) is one of the biggest commercial farms in Zimbabwe. During the study period, the two herds were under similar management and feeding regimens. Animals were fed on pasture during the wet season but were fed with chopped *Zea mays* silage and on-farm produced grass hay in stalls with limited access to irrigated pasture during the dry season. During milking sessions, lactating cows were each fed a 2 kg total mixed ration comprising snapped corn mixed with dairy concentrate formulations from Agrifoods Ltd, Harare, Zimbabwe. Crude protein content in these commercial feeds varied from 19% for cows during early lactation, 17% during mid-lactation, 15% during late lactation and 12% during the dry period. Metabolisable energy (MJ ME/kg) varied from 11.5 for cows during early lactation, 11.0 during mid-lactation, 10.5 during late lactation and 9.0 during the dry period. Calcium varied from 0.80% for cows in early lactation, 0.70% during mid-lactation, 0.65% during late lactation and 0.60% during the dry period. Total withdrawal of calcium pre-calving was never practised in either herds. There was no strict adherence by the farm management to an optimum body condition of 3.5 pre-calving. Because of the high numbers of animals and scarcity of pre-calving housing space on this farm, heifers and mature cows were not housed separately prior to calving. An independent nutritional analysis was not performed during this study. All the health and production records used in this study were kept and maintained by the farm manager with the assistance of the farm veterinarian. Herd health and production records were kept on-site, and the data collected included breed, parity, calving date, date of occurrence of milk fever and 305-day total milk production per individual cow. As some animals had more than one lactation in the 3 years of study, a total of 1278 parturitions were studied. Clinical diagnosis of milk fever was carried out by the farm para-veterinary staff and veterinarians on the basis of clinical signs that included initial weakness (characterised by ataxia) and trembling with spasmodic localised muscle contractions, followed by tachycardia and mild hyperthermia associated with tetany in the early stages. Muscular weakness progressing into sternal and occasionally lateral recumbency was acceptably more consistent clinical signs of milk fever. Weak pulse, dilated pupils and rumen atony with mild to severe bloating were other signs indicative of milk fever. Diagnoses suggested by the para-veterinary staff were always confirmed by the veterinarian, following which calcium borogluconate was intravenously administered. Because plasma calcium concentrations were never investigated, confirmation of milk fever was accomplished by response to treatment with calcium borogluconate. For the purpose of this study, only cows diagnosed with stage 2 of milk fever, that is, the recumbent stage, were included in the investigation. No repeat cases of milk fever were encountered.

### Trustworthiness

All the data used in this study were collected by two farm managers, four para-veterinary staff and three veterinarians. Examinations were performed in accordance with animal welfare as well as occupational health and safety guidelines as defined by Zimbabwe Veterinary Services. Therefore, all the results presented in this article are trustworthy.

## Results

Results in Table 1 show that Jersey cows had a significantly higher incidence of milk fever than Holstein cows (*p* < 0.05).

**TABLE 1:** Incidence of milk fever according to breed of cow.

Breed of cow	Total number of parturitions	Number of milk fever cases	Incidence of milk fever (%)
Jersey	406	60	14.78*
Holstein	872	42	4.82*
Overall	1278	102	7.98

* Chi-square value = 37.43; *p* = 0.00; *N* = 1278. Difference was significant because *p* < 0.05.

Results in Table 2 show that the incidence of milk fever in Jersey cows with more than four parities was significantly higher than those in their second, third and fourth parities (*p* < 0.05). There was, however, no significant difference in the incidence of milk fever between Jersey cows in their second, third and fourth parities (*p* > 0.05).

**TABLE 2:** Incidence of milk fever according to parity in Jersey cows.

Parity of cow	Total number of parturitions	Number of milk fever cases	Incidence of milk fever (%)
2	34	2	5.90
3	77	5	6.49
4	126	11	8.73
> 4	169	42	24.85
Overall	406	60	14.78

Results in Table 3 show that the incidence of milk fever in Holstein cows with more than four parities was significantly higher than those in their second, third and fourth parities (*p* < 0.05). There was, however, no significant difference in the incidence of milk fever between Holstein cows in their second, third and fourth parities (*p* > 0.05).

**TABLE 3:** Incidence of milk fever according to parity in Holstein cows.

Parity of cow	Total number of parturitions	Number of milk fever cases	Incidence of milk fever (%)
2	70	1	1.43
3	165	3	1.82
4	275	8	2.91
> 4	362	30	8.29
Overall	872	42	4.82

Results in Table 4 show that Jersey cows producing more than 6114 litres per 305-day lactation had significantly higher incidence of milk fever than those producing less than 6114 litres per 305-day lactation (*p* < 0.05). There was, however, no significant difference in the incidence of milk fever in Jersey cows in the first, second and third quartiles of milk production (*p* > 0.05).

**TABLE 4:** Incidence of milk fever according to milk yield in Jersey cows.

Total of milk production per 305-day lactation (L)	Total number of parturitions	Number of milk fever cases	Incidence of milk fever (%)
< 3975	29	2	6.90
3975–4827	102	8	7.84
4828–6114	142	14	9.86
> 6114	133	36	27.07
Overall	406	60	14.78

Results in Table 5 show that Holstein cows producing more than 9149 litres per 305-day lactation had significantly higher incidence of milk fever than those producing less than 9149 litres per 305-day lactation (*p* < 0.05). There was, however, no significant difference in the incidence of milk fever in Holstein cows in the first, second and third quartiles of milk production (*p* > 0.05).

**TABLE 5:** Incidence of milk fever according to milk yield in Holstein cows.

Total of milk production per 305-day lactation (L)	Total number of parturitions	Number of milk fever cases	Incidence of milk fever (%)
< 6324	62	1	1.61
6324–7542	219	3	1.37
7543–9149	305	8	2.62
> 9149	286	30	10.49
Overall	872	42	4.82

## Ethical considerations

During the period of study, conventional dairy animal husbandry was adhered to and no animals were mistreated, starved or subjected to unorthodox procedures or treatments. Researchers and farm staff strictly observed animal welfare and public health guidelines.

## Discussion

The incidence of milk fever reported in both the Jersey and Holstein herds during this study (7.98%) was within the 5% – 10% range reported by Houe et al. (2000) and later by Roche and Berry (2006). The incidence rate was, however, higher than the 3.45% and 3.50% reported in 10 American and Australian studies, respectively, and moderately higher than the 6.17% reported in European studies (DeGaris & Lean 2008). According to Mulligan and Doherty (2008), the typical incidence rate of milk fever varies between 3.5% and 7.0%. This may be true for the West and may not be for Africa. Possibly the lower figures in Western world could be attributed to improvements in feed management practices and better climatic adaptations for these breeds. It is, however, difficult to reconcile these differences given that Western herds produce more milk than Zimbabwean herds. Climatic conditions have been identified as possible factors influencing the incidence of metabolic disorders. The difference in the amount of UV radiation (Damgaard 1975), average evaporation rate and the difference between maximum and minimum ambient temperature (Capper & Cady 2012; Roche & Berry 2006) in Zimbabwe and other countries studied may significantly affect the frequency of milk fever.

Results of this study demonstrated that breed (Table 1), parity (Tables 2 and 3) and level of milk production (Tables 4 and 5) were significantly associated with the occurrence of milk fever. The higher occurrence of milk fever in Jerseys (14.78%) compared with Holsteins (4.82%) observed in this study (Table 1) supports earlier findings (Lean et al. 2006; Roche & Berry 2006). Goff (2008) attributed this difference to reduced concentrations of intestinal receptors for calcitriol in Jersey cattle compared with Holsteins. Dairy management systems have been reported to focus mainly on providing feed formulations aimed in maximum milk output and fertility (Mulligan et al. 2006b). However, because of lack of research comparing the digestive physiology and nutrition of modern Jersey and Holstein cows, current guidelines for feeding dairy cows have reportedly failed to make specific recommendations for Jerseys (Aikman, Reynolds & Beever 2008). Aikman et al. (2008) pointed out that because of differences in nutritional requirements and digestive capacity of the two breeds, especially during the periparturient period (3 weeks pre-parturition and 1 week post-partum), Holstein-derived feed formulations were not wholly appropriate for Jersey diets. Hence, this discrepancy is believed to make the Jerseys vulnerable to various nutritional deficiencies and subsequently more susceptible to milk fever.

The significant association of increased milk fever occurrence with an increase in parity observed in this study (Tables 2 and 3) agrees with earlier observations (Horst 1986; Krehbiel 2014). This association is reportedly because of the reduced ability to mobilise calcium from bones, a decline in intestinal transport of calcium and the reduced ability to produce calcitriol in older cows (Horst 1986). Results of this study also demonstrated the influence of the level of milk production on the occurrence of milk fever (Tables 4 and 5). High levels of milk production have reportedly been translated to mean higher loss of calcium through milk (Horst 1986) and have hence led to higher occurrence of milk fever.

The risk of milk fever is higher in Jerseys compared with Holsteins (Lean et al. 2006), especially those in their fourth parities and with production levels within the fourth quartile. This information can be used to better protect at-risk cows in grazing systems through increased vigilance by herd personnel and implementation of additional control strategies during high-risk periods for at-risk cows. The researchers propose that the farm management would benefit from feeding calcium-deficient diets in the late dry period, feeding a calcium-rich diet 3–4 days before parturition, vitamin D supplementation, reducing the dietary cation–anion difference and magnesium supplementation in the late gestation period as suggested by Goff (2008) and Mulligan et al. (2006b). However, prior nutritional analysis of the various feeds provided for the animals on this farm would be required to effectively implement the proposed dietary manipulations to avoid upsetting the nutritional balance. In addition, strict adherence to an optimum body score condition of 3.5 pre-calving (Amral-Phillips 2014) is also suggested for the farmer. According to the same author, reduction of stress in close-up dry calves by creation of separate housing for heifers and mature cows pre-calving has been shown to reduce the incidence of milk fever and is hereby recommended at this farm. This study also supports the view by Lean et al. (2006) that Holsteins and Jerseys should not be kept under identical management conditions. Further research is necessary to compare the two breeds and other dairy breeds found in Zimbabwe under different management systems.
